# The Stir Sequence in MRI of Neoplastic Lesions

**Published:** 1988-05

**Authors:** P. Goddard, J. Waring, A. Case, R. Yeats, J. A. Bullimore, E. Whipp, V. Barley


### The Stir Sequence in MRI of Neoplastic Lesions

**P. Goddard MD, FRCR, J. Waring, A. Case, R. Yeats, J. A. Bullimore FRCR, E. Whipp FRCR, V. Barley FRCR**

### INTRODUCTION

The Short Tau Inversion Recovery or STIR sequence was devised by Drs Bydder and Young of the Hammersmith Hospital to enhance lesions and suppress surrounding fat (1). Experience with seventy patients studied with suspected neoplastic conditions has shown that the use of the STIR sequence increases the sensitivity of MRI scanning. It also has the potential to decrease scanning and reporting time (2).

### Method

Seventy patients with suspected neoplastic disease were studied using a Picker Vista 2055 HP 0.5 T Magnetic Resonance Scanner. The STIR sequence was used in all patients in addition to spin echo sequences and the signal level of lesions compared with surrounding tissues was assessed visually.

### Results

The results are shown in the Table. The STIR sequence showed a high signal intensity (white) in all malignant diseases studied. These included a large variety of carcinomas (including bronchus and breast), lymphoma, sarcoma and teratoma.

In benign disease the results were more variable with a high signal in lesions such as angiomata and a-v malformations and low signal (dark) in lipoma. An unusual, characteristic pattern was shown in uterine fibroids with a predominantly low signal mass with high signal septa or striation.

High signal was also shown on STIR images of abscesses and of normal structures such as the spleen, kidneys, gut wall and seminal vesicles.

The STIR sequence provides very good contrast between aggressive lesions and normal tissues such as fat, muscle, liver and fatty marrow.

Examples of the use of the STIR sequence are included in the papers in this issue on the detection of metastatic disease in the lumbar spine and in the pelvis.

### Conclusion

The STIR sequence is extremely valuable in identifying areas of abnormality that could otherwise be missed on other sequences and in delineating the extent of abnormal tissue.

| | Number of Patients | STIR appearance |
|---|---|---|
| Benign Tumours | | |
| Lipoma | 4 | Very low signal |
| Uterine leio-myo-fibroma | 2 | Mainly low signal with high signal septa |
| Angioma | 2 | Very high signal |
| Malignant Tumours | | All high signal |
| Carcinoma: | | |
| Bladder | 8 | |
| Prostate | 10 | |
| Cervix | 8 | |
| Bronchus | 7 | |
| Breast | 5 | |
| Antrum | 3 | |
| Rectum | 2 | |
| Vagina | 2 | |
| Uterus | 1 | |
| Lymphoma | 3 | |
| Liposarcoma | 2 | |
| Multiple myeloma | 1 | |
| Mesothelioma | 1 | |
| Teratoma | 3 | |
| Rhabdomyosarcoma | 2 | |
| Uveal melanoma | 1 | |
| Glioma | 2 | |
| Chondrosarcoma | 1 | |

### REFERENCES

1. BYDDER, G. M. and YOUNG, I. R. (1985) "MR Imaging: Clinical use of the Inversion Recovery Sequence". *J. Comput Assist Tomogr*, **9**, 659–675
2. SUE NELSON (1987) "Clinical Advantages of STIR Imaging Vs. Conventional T2 Spin Echo Methods" *NVR (Picker Int.)* Vol 2, No. 3, pp 18–21.

